# Graph theory analysis of resting‐state functional magnetic resonance imaging in essential tremor

**DOI:** 10.1002/hbm.24900

**Published:** 2019-12-15

**Authors:** Constantin Tuleasca, Thomas Bolton, Jean Régis, Tatiana Witjas, Nadine Girard, Marc Levivier, Dimitri Van De Ville

**Affiliations:** ^1^ Assistance Publique‐Hôpitaux de Paris Hôpitaux Universitaires Paris‐Sud, Centre Hospitalier Universitaire Bicêtre, Service de Neurochirurgie Paris France; ^2^ Faculté de Médecine Sorbonne Université Paris France; ^3^ Centre Hospitalier Universitaire Vaudois (CHUV) Neurosurgery Service and Gamma Knife Center Lausanne Switzerland; ^4^ Medical Image Analysis Laboratory (MIAL) and Department of Radiology‐Center of Biomedical Imaging (CIBM) Centre Hospitalier Universitaire Vaudois Lausanne Switzerland; ^5^ Signal Processing Laboratory (LTS 5) École Polytechnique Fédérale de Lausanne (EPFL) Lausanne Switzerland; ^6^ Faculty of Biology and Medicine University of Lausanne Lausanne Switzerland; ^7^ Medical Image Processing Laboratory École Polytechnique Fédérale de Lausanne (EPFL) Lausanne Switzerland; ^8^ Faculty of Medicine University of Geneva Geneva Switzerland; ^9^ Stereotactic and Functional Neurosurgery Service and Gamma Knife Unit CHU Timone Marseille France; ^10^ Neurology Department CHU Timone Marseille France; ^11^ Department of Diagnostic and Interventionnal Neuroradiology, Faculté de Médecine et APHM, Hôpital Timone AMU, CRMBM UMR CNRS 7339 Marseille France

Dear Editor,

1

We read with great interest the recent article of Benito‐Leon et al. (2019). The authors acquired resting‐state functional magnetic resonance imaging (fMRI) data in patients with essential tremor (ET), and compared them to healthy controls (HC). Specifically, they used graph theory analysis to assess functional network organization. Interestingly, widespread brain regions outside the classically described “cerebello‐thalamo‐cortical” axis (also known as the *tremor network*) are reported as being disrupted. Patients showed significantly higher values of global efficiency, cost and degree, and a shorter average path length in the left inferior frontal gyrus (pars opercularis), right inferior temporal gyrus (posterior division and temporo‐occipital part), right inferior lateral occipital cortex, left paracingulate, bilateral precuneus bilaterally, left lingual gyrus, right hippocampus, left amygdala, nucleus accumbens bilaterally, and left middle temporal gyrus (posterior part). In addition, significant higher local efficiency and clustering coefficient values in frontal medial cortex bilaterally, subcallosal cortex, posterior cingulate cortex, parahippocampal gyri bilaterally (posterior division), right lingual gyrus, right cerebellar flocculus, right postcentral gyrus, right inferior semilunar lobule of cerebellum and culmen of vermis were found in ET. Finally, the right intracalcarine cortex and the left orbitofrontal cortex showed a shorter average path length in ET patients, while the left frontal operculum and the right planum polare showed a higher betweenness centrality in ET patients.

There are a number of key points that caught our attention and would deserve further clarifications. First, in the *Introduction*, the authors state: “only a few studies have explored connectivity alterations in ET, and those that did were mainly focused in the cerebello‐thalamo‐cortical network (…) or on the study of surgical therapeutic applications”. Benito‐Léon et al. (Benito‐Leon et al., 2019) cite one of our previous studies (following also a reference of Akram et al. (2018)), which analyzed rs‐fMRI data before (pretherapeutically) and after thalamotomy for ET (Tuleasca, Regis, Najdenovska, et al., 2018b). We used seed‐based functional connectivity (FC) to characterize time courses of the motor thalamus. Initially, motor thalamus has been segmented individually, for each patient, using pretherapeutic diffusion‐weighted imaging (DWI), as part of a larger neuroimaging protocol (T1 weighted, rs‐fMRI, DWI). This segmentation was performed using an in‐house methodology (Najdenovska et al., 2018) and the Morel atlas as reference (Morel, Magnin, & Jeanmonod, 1997). Furthermore, the individual motor thalamus (obtained from DWI) time‐courses were extracted from rs‐fMRI. However, our study focused not only on therapeutic implications of thalamotomy of the ventro‐intermediate nucleus (Vim) in terms of difference in FC before and after the intervention, but also on the comparison between pretherapeutic ET and HC. As an example, pretherapeutic standard tremor scores were correlated in a statistically significant way with FC between ventral‐lateral ventral thalamus (VLV according to Morel nomenclature) and the primary sensory‐motor area, but also with other regions, such as the pedunculopontine nucleus. We reported changes in FC 1 year after thalamotomy in right insular and orbitofrontal cortex, supramarginal gyrus, anterior insula, or the inferior frontal gyrus, parts of which are also reported by Benito‐Leon et al. (2019). We postulated that the commonly targeted ventrolateral thalamus for drug‐resistant ET would act as a mediator after the intervention, inducing major changes in dorsal attention, salience, and supplementary motor networks. The insula would act as a hub, in downregulating the relationship between all these aforementioned, structurally segregated, yet functionally highly interconnected systems. Moreover, using the same methodology (i.e., seed‐based approach) and in a second study, we coined the term *cerebello‐thalamo‐visuo‐motor network* (Tuleasca, Regis, Najdenovska, et al., 2018a) to describe the fact that pretherapeutic VLV FC with right visual association area (Brodmann area, BA19) predicted 1‐year activities of daily living (ADL; Bain et al., 1993) decrease. This revelation of visual areas implicated in ET followed other publications from our group, on the same topic, on both functional (please see below) and structural aspects (Tuleasca, Witjas, Najdenovska, et al., 2017; Tuleasca, Witjas, van de Ville, et al., 2017).

Second, still in the *Introduction*, Benito‐Léon and co‐authors state: “Neither seed‐based functional connectivity nor the independent component analysis approach, which are the most widely used rs‐fMRI analysis techniques, can completely characterize the brain functional network (…), which is, in turn, dynamic, as it provides support for several cognitive and emotional processes (…) that might be altered in ET”. In light of the above, Benito‐Léon and co‐authors propose using other approaches. Although graph theory analysis has evident interpretational merits, as detailed by the authors, we wish to emphasize that it does not permit to gain any dynamic insight in terms of brain function in the form deployed by the authors: indeed, FC remains computed on full rs‐fMRI time courses, as in the case of seed‐based functional connectivity. A dynamic view could only be gained if graph metrics were instead iteratively computed on temporal sub‐windows of data. Other approaches of interest in the context of rs‐fMRI in ET patients enable to overcome this limitation of the classical seed‐based methodology. In fact, we recently deployed co‐activation pattern (CAP) analysis (Tuleasca et al., 2019), which allows investigating how a specific seed region connects with the rest of the brain in time‐varying fashion (Liu & Duyn, 2013). To explore this, we assessed a subpart of the right extrastriate cortex (Brodmann area 19—including V3, V4, and V5) as a unique region of interest (ROI). This seed was chosen from our previously published data (Tuleasca, Najdenovska, Regis, et al., 2018a; Tuleasca, Regis, Najdenovska, et al., 2018b), and further confirmed by task‐based studies (Archer et al., 2017), given its FC with the cerebellum lobule VI, bilateral motor cortex and frontal eye fields in ET. We generated the different whole‐brain network patterns (CAPs, Figure 1, left; Tuleasca et al., 2019) that occur over time when this ROI is active on a group consisting of both HC and pretherapeutic ET patients. We further obtained the occurrences of each CAP, and correlated them with tremor severity, using clinically relevant standard tremor scores. We found three different CAPs, comprising “cerebello‐visuo‐motor” (CAP 1), “thalamo‐visuo‐motor” (CAP 2, including the targeted thalamus) and “basal ganglia and extrastriate cortex” networks (CAP 3). The occurrence of the first CAP was decreased in pretherapeutic ET as compared to HC, while the other two CAPs showed increased occurrences (Figure 1, right). All CAPs had their occurrences normalize back to HC level after the intervention (Figure 1). Multiple regression analysis showed that pretherapeutic ET standard tremor scores negatively correlated with increased occurrence of the thalamo‐visuo‐motor (CAP 2) network (Figure 2, left). Clinical improvement after thalamotomy was related to changes in occurrences of the basal ganglia and extrastriate cortex circuitry (Figure 2, right), suggesting a role of dynamics of the extrastriate cortex in tremor generation and further arrest after the intervention.

**Figure 1:**
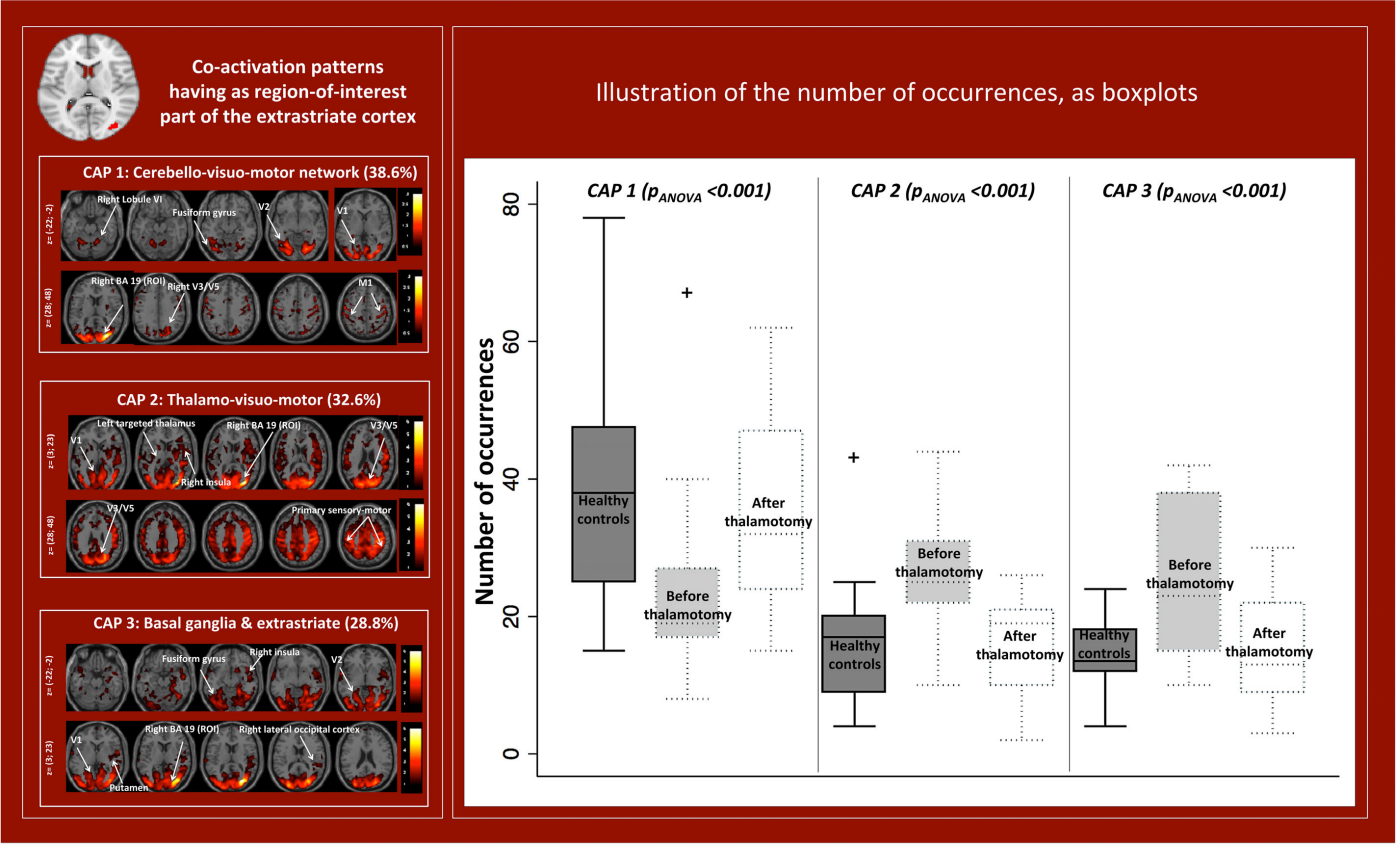
Illustration of the three CAPs (left), from 1 to 3, as well as the corresponding number of occurrences (left), as boxplots, for each CAP, in HC, pretherapeutic, and posttherapeutic ET states (adapted from Tuleasca et al., 2019)

**Figure 2:**
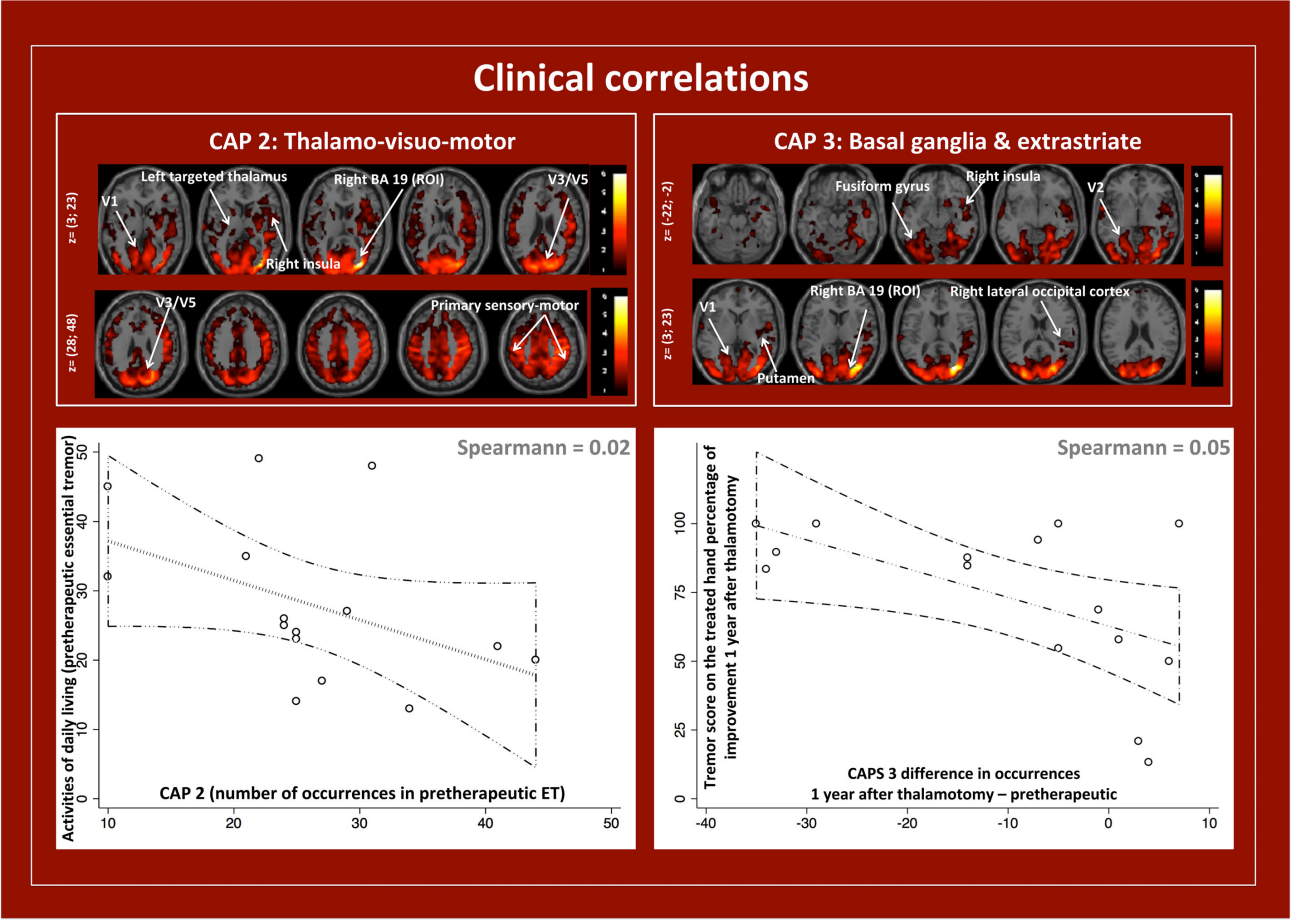
Clinical correlation between (left) pretherapeutic activities of daily living with the number of occurrences for CAP_2_ and (right) posttherapeutic tremor score on the treated hand and difference in occurrences between the posttherapeutic and pretherapeutic states for CAP_3_ (adapted from Tuleasca et al., 2019)

A third point relates to the *Discussion* section: “The lack of correlation between any graph theory metric and tremor duration or severity merits an explanation“. This is most probably the most intriguing point of the present study of Benito‐Léon and co‐authors (Benito‐Leon et al., 2019). This deserves further explanations but remains a major drawback. The authors state “overall tremor duration is not a reliable variable since differences in reported age of onset in ET may vary widely (…). With respect to severity, one should keep in mind that the Fahn‐Tolosa‐Marin tremor rating scale is a motor scale (…) and most of the observed intergroup differences in connectivity were found in extra‐motor areas.” If one uses graph theory to overcome limitations of other approaches (including ICA and seed‐based FC), but no clinical correlation is found, the present report remains a purely descriptive neuroimaging one. Moreover, other authors have described clinically meaningful correlations for regions outside the cerebello‐thalamo‐cortical network, which appear altered in ET. In fact, using task‐based fMRI, Archer et al. (2017) provided evidence of a widespread visually sensitive functional network, including extrastriate areas V3 and V5, that relates to tremor severity in patients with essential tremor (ET). The authors were able to obtain right‐hand force measurements during functional MRI of a grip‐force task while they manipulated visual feedback. They concluded that the severity of tremor is exacerbated by increased visual feedback (Archer et al., 2017). We used data‐driven multivariate analysis (i.e., independent component analysis; Beckmann, DeLuca, Devlin, & Smith, 2005; Calhoun, Adali, Pearlson, & Pekar, 2001) to conduct whole‐brain analysis without prior assumptions (Tuleasca, Najdenovska, Regis, et al., 2018b) on rs‐fMRI data in ET, before and after thalamotomy. We found two networks, which reflected the interaction between time (pretherapeutic and 1 year post‐thalamotomy) and clinical effect; this included the bilateral motor network, frontal eye‐fields and left cerebellum lobule VI (the former like in the report of Archer et al. (2017)), of which network interconnectivity strength with right visual BA 19 related to tremor arrest after thalamotomy of the Vim (Tuleasca, Najdenovska, Régis, et al., 2018a); another network was the salience network, involving bilateral superior frontal gyri and insular areas (c9) with left claustrum and putamen. Moreover, while evaluating the time effect independently of the clinical one, a component reminiscent of the salience network showed altered interconnectivity with right fusiform gyrus and V5. We suggested that relevant networks in ET are visual, motor, and attention ones (Tuleasca, Najdenovska, Regis, et al., 2018a). Multiple differences were found between patients who alleviated less and those who alleviated more after thalamotomy, independently of time‐point (pretherapeutic ET vs. posttherapeutic), involving the following relevant anatomical regions: cerebellum (comprising the dentate nucleus), basal ganglia including the thalamus (both right and left) and putamen, frontal lobe (encompassing frontal eye fields and the dorsolateral prefrontal cortex), parietal lobe (angular and supramarginal gyrus), temporal lobe (middle temporal area), occipital lobe (visual association areas, retrosplenial cortex), cingulate (both anterior dorsal and posterior), part of them being also reported by Benito‐Leon et al. (2019).We agree with the authors that there is clinical heterogeneity in ET and that some subgroups of patients seem to differ from others, including those with head tremor. In fact, we performed such analysis on 11 cases with head tremor, in the frame of ET, pretherapeutically, as compared with HC, using ICA. We postulated that the supplementary motor area is modulating head tremor appearance, by abnormal connectivity with the thalamolimbic system (Tuleasca, Regis, Najdenovska, et al., 2018a).

In addition to the above points, we would also like to draw attention to two other aspects. One is related to motion as a potential confounding factor in this particular clinical population. We have systematically computed Power's framewise displacement index for each time point (Power, Barnes, Snyder, Schlaggar, & Petersen, 2012) in all our patients and had to exclude several of them from our perspective clinical and neuroimaging protocol, so as to avoid obtaining spurious correlations which could have potentially appeared after our analysis, in the absence of such caution. In fact, our rs‐fMRI data consisted of 300 volumes of a repeated gradient echo‐planar imaging T2*‐weighted sequence (as opposed to only 120 in the study of Benito‐Léon and co‐authors) and we reported that pretherapeutically, the mean number of frames taken out was 35 (median 15, range 0–135), and at 1 year after thalamotomy, was also 35 (median 15, range 0–150) (Tuleasca, Najdenovska, Regis, et al., 2018d). Here (Benito‐Leon et al., 2019), although the authors report that data points associated with too large instantaneous motion were not included in their analyses (through the use of dedicated scrubbing repressors as covariates), there is no mention of the framewise displacement threshold that was used in doing so. In addition, it is also not reported how many frames before and after an event of excessive motion were excluded from the analyses, there is no mention of discarding any subject due to too many frames being corrupted, and there is no comparison between the healthy and ET groups in terms of the in‐scanner motion.

A last aspect is related to the scanning of these patients under medication. Out of the 23 ET patients, “15 (65.2%) were taking one or more anti‐tremor medications (six propranolol, one primidone, one clonazepam, one clonazepam and propranolol, one gabapentin and primidone, one primidone and propranolol, one zonisamide and primidone, one zonisamide and propranolol, one zonisamide, primidone, and alprazolam, and one zonisamide, primidone, and propranolol)”. Drug influence on resting‐state fMRI data is now a fact that is proven, considered relevant in such studies. In fact, medication can change the network's status and can influence the blood oxygenation level‐dependent (BOLD) signal (Wandschneider & Koepp, 2016).

The study of Benito‐Leon et al. (2019) is interesting, as it confirms some of the previously published research on ET patients, using both rs‐fMRI or task‐based fMRI, while describing network abnormalities outside the classical “tremor axis.” Despite the absence of clinical correlations, this exploratory research is still interesting, as it confirms new promising avenues for research and contributes to new hypotheses. Nevertheless, we would encourage the authors and other colleagues in the field to be thoughtful when reporting and/or interpreting the study results, especially related to the motion analysis, drug noninterruption issues and the absence of clinical correlation scores. Further strengthening studies with respect to these aspects will lead to increased clinical impact.

## Data Availability

Data sharing is not applicable to this article as no new data were created or analyzed in this study.
